# PReS-FINAL-2146: Trends in prescription of biologics and outcomes of juvenile idiopathic arthritis; results of the Dutch national arthritis and biologicals in children register

**DOI:** 10.1186/1546-0096-11-S2-P158

**Published:** 2013-12-05

**Authors:** MH Otten, J Anink, FH Prince, M Twilt, SJ Vastert, R Ten Cate, EP Hoppenreijs, W Armbrust, SL Gorter, PA Van Pelt, S Kamphuis, KM Dolman, JF Swart, JM Van den Berg, Y Koopman-Keemink, MA Van Rossum, NM Wulffraat, LA Van Suijlekom-Smit

**Affiliations:** 1 Erasmus MC Sophia Children's Hospital, Rotterdam, Netherlands, Netherlands; 2 Birmingham Children's Hospital, Birmingham, UK; 3 University Medical Centre Utrecht, Utrecht; 4 Leiden University Medical Centre, Leiden, Netherlands; 5 St Maartenskliniek and Radboud University Nijmegen Medical Center, Nijmegen, Netherlands; 6 University Medical Centre Groningen, Groningen, Netherlands; 7 University Medical Centre Maastricht, Maastricht, Netherlands; 8 St. Lucas Andreas Hospital and Reade Institute, Amsterdam, Netherlands; 9 Academic Medical Centre and Reade Institute, Amsterdam, Netherlands; 10 Hagaziekenhuis Juliana Children's Hospital, The Hague, Netherlands

## Introduction

Treatment of juvenile idiopathic arthritis (JIA) has changed dramatically since the introduction of biologics in 1999. Because of more insight in the cytokines involved in JIA the number of available biologic agents increased. Together with the introduction of these new drugs, new insights in the optimal treatment of JIA indicate that earlier and more aggressive therapy is associated with better outcomes. Whether these developments with regard to biologic treatment have resulted in better patients' outcomes in daily practice is not yet reported.

## Objectives

To evaluate trends in prescription of biologics and influence on outcomes of Dutch JIA patients that started their first biologic between 1999 and 2010.

## Methods

The Arthritis and Biologicals in Children register (a multicenter prospective observational study) aims to include all JIA patients in the Netherlands who use or used biologic agents since 1999. Patients were divided in time periods according to the year of introduction of first biologic agent. Trends in characteristics of patients before introduction of first biologic and effectiveness of the first biologic were analyzed over a 12 year period.

## Results

343 non-systemic and 86 systemic JIA patients started at least 1 biologic agent between 1999 and 2010. Etanercept remained biologic of first choice for non-systemic JIA and anakinra has become first choice for systemic JIA. The use of systemic prednisone and synthetic disease-modifying anti-rheumatic drugs (besides methotrexate) prior to biologics decreased. During these 12 years of observation, biologics were prescribed after shorter disease durations; the proportion of patients with less than 1.5 years of disease duration before start of the first biologic agent increased from zero in the years 1999-2001 to 31% in 2008-2010. Disease activity and acquired sequelae at baseline decreased with regard to number of joints with arthritis (median of 18 in 1999-2001 decreased to 5 in 2008-2010), number of joints with limited motion (median of 12 in 1999-2001 decreased to 3 in 2008-2010) and functional disability (CHAQ) scores (median score of 1.8 in 1999-2001 decreased to 1.1 in 2008-2010). In systemic JIA biologics are now being introduced in patients with higher ESR values. These changes resulted in more patients with inactive disease and less joints with limited motion after 3 and 15 months of treatment in all JIA categories.

## Conclusion

Biologics are prescribed increasingly, are introduced earlier during the disease course and in JIA patients with lower disease activity. These changes are accompanied by better short-term disease outcomes. Etanercept remains biologic of first choice for non-systemic JIA and anakinra has become first choice for systemic JIA.

## Disclosure of interest

M. Otten Grant/Research Support from: Abbott, Novartis, Roche, Pfizer, Consultant for: Roche, J. Anink: None declared., F. Prince: None declared., M. Twilt: None declared., S. Vastert Consultant for: Novartis, R. Ten Cate Grant/Research Support from: Pfizer, Consultant for: Pfizer, E. Hoppenreijs: None declared., W. Armbrust: None declared., S. Gorter: None declared., P. Van Pelt: None declared., S. Kamphuis Grant/Research Support from: Pfizer, glaxosmithkline, K. Dolman: None declared., J. Swart: None declared., J. Van den Berg: None declared., Y. Koopman-Keemink: None declared., M. Van Rossum: None declared., N. Wulffraat Grant/Research Support from: Pfizer, Novartis, Roche, abbvie, L. Van Suijlekom-Smit Grant/Research Support from: Dutch Board of Health Insurances, Dutch Arthritis Association, Pfizer, Abbott, Consultant for: Roche, Novartis.

